# Temporal-Quality Ensemble Technique for Handling Image Blur in Packaging Defect Inspection

**DOI:** 10.3390/s24144438

**Published:** 2024-07-09

**Authors:** Guk-Jin Son, Hee-Chul Jung, Young-Duk Kim

**Affiliations:** 1 ICT Research Institute, Daegu Gyeongbuk Institute of Science and Technology, Daegu 42988, Republic of Korea; sudopop@dgist.ac.kr; 2 Department of Artificial Intelligence, Kyungpook National University, Daegu 41566, Republic of Korea; heechul@knu.ac.kr

**Keywords:** packaging, defect inspection, deep learning, image blur, ensemble, temporal-quality analysis

## Abstract

Despite achieving numerous successes with surface defect inspection based on deep learning, the industry still faces challenges in conducting packaging defect inspections that include critical information such as ingredient lists. In particular, while previous achievements primarily focus on defect inspection in high-quality images, they do not consider defect inspection in low-quality images such as those containing image blur. To address this issue, we proposed a noble inference technique named temporal-quality ensemble (TQE), which combines temporal and quality weights. Temporal weighting assigns weights to input images by considering the timing in relation to the observed image. Quality weight prioritizes high-quality images to ensure the inference process emphasizes clear and reliable input images. These two weights improve both the accuracy and reliability of the inference process of low-quality images. In addition, to experimentally evaluate the general applicability of TQE, we adopt widely used convolutional neural networks (CNNs) such as ResNet-34, EfficientNet, ECAEfficientNet, GoogLeNet, and ShuffleNetV2 as the backbone network. In conclusion, considering cases where at least one low-quality image is included, TQE has an F1-score approximately 17.64% to 22.41% higher than using single CNN models and about 1.86% to 2.06% higher than an average voting ensemble.

## 1. Introduction

Packaging is widely utilized in logistics and transportation [1] but typically suffers from diverse surface defects stemming from external forces and repetitive compression. These defects of packaging cause significant damage or deterioration of the product inside. Moreover, it is also critical when important information like ingredient lists on packaging surface labels gets damaged. Traditionally, the defect inspection of packaging surfaces was usually performed by manual inspection. However, manual inspection is slow, inefficient, and has a high error rate in finding defects [2] in large volumes of packaging. To replace manual inspection, several studies were conducted for surface defect inspection using computer vision [3,4,5]. Nevertheless, these existing methods are not widely used in inspection industries due to a lack of generalization, robustness, and accuracy.

Recently, deep learning [6] has received a lot of attention for the inspection of surface defects [7,8,9,10]. Deep learning models can identify the unique features of packaging and their defects by training on datasets. However, when relying solely on convolutional neural network (CNN)-based architecture, factors such as motion blur [11] occurring in packaging-related industrial environments cannot be forecasted in advance during the training of the model. Regarding low-quality images, packaging-related industrial environments often face two types of low-quality images: motion blur caused by conveyor movement; and focus blur, known as the out-of-focus phenomenon.

**Figure 1:**
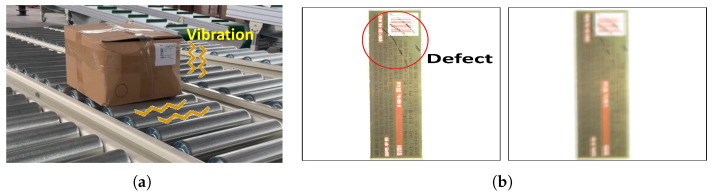
Challenges in conveyor systems and packaging defect inspection. (**a**) Vibration with roller conveyor. (**b**) Out of focus with machine vision.

First, the main causes of motion blur are as follows. Although the conveyor contributes to the movement of packaging, it often generates significant vibrations when foreign substances adhere to the packaging or when the exposed surface of the conveyor belt is worn or damaged. Additionally, roller-based conveyors, which are commonly used in warehouses, can also cause vibration. This is shown in Figure 1a. This vibration of the conveyor temporarily increases the moving speed of the packaging product, and relatively decreases the camera shutter speed, which results in motion blur [12,13,14,15,16]. Second, focus blur mainly occurs when a camera or lens with an autofocus function changes the brightness level or loses focus [17,18]. For example, focus blur occurs when packaged products on a conveyor belt have different brightness levels due to changing lighting or when the focus moves irregularly from one packaged product to the next one. Figure 1b shows an example of an out-of-focus problem. These blur problems are not only the main causes of producing low-quality images but also degrade the performance of CNN models. To this end, adopting ensemble techniques that refer to images from various timing captured images can be a solution to mitigate these issues.

Many academics have presented research data over the last decade indicating that ensemble approaches outperform single classifiers [19,20]. Ensemble learning is a machine learning technique that combines multiple models to obtain better prediction performance, typically by aggregating their predictions [21]. Among them, average voting ensemble (AVE) [22] is a popular ensemble learning strategy [23] which provides the most common prediction among the models as the final output. However, when there is a mixture of low-quality (e.g., blur) and high-quality images, AVE suffers from performance degradations because low-quality images have a negative impact on prediction by allocating equal weight to all images with different qualities.

To address this challenge, we propose a noble inference technique named temporal-quality ensemble (TQE) which integrates information from multiple images with different qualities. For this, it consists of two key components: quality weight and temporal weight. Quality weight evaluates individual frame quality and then prioritizes high-quality images in order to ensure the inference process emphasizes reliable information while it deprioritizes low-quality images. Temporal weight accounts for the temporal continuity and assigns weights to images based on their timing and relation to observed images. This approach, which emphasizes the importance of up-to-date images, has the effect of balancing the quality weights without simply excluding important low-quality images that may contain important information about defects.

The main contributions of this paper are summarized as follows.

(1) The proposed inference technique, termed TQE, combined temporal and quality weight to integrate information from multiple images including blurred images. By leveraging temporal continuity and prioritizing superior clarity, it finally mitigates the effects of image blur and improves overall accuracy for identifying defects. To the best of our knowledge, the proposed approach is the first ensemble technique to overcome image blur for packaging inspection.
(2) Our new private database provided more realistic results for training and evaluating deep learning models since it reflected motion blur in images which are acquired by deploying a real machine vision camera and conveyor belt, etc.
(3) Through comparative experiments with AVE, TQE exhibited effectiveness in terms of accuracy, precision, recall, and F1-Score for identifying defects.

## 2. Related Work

Surface defect inspection often uses CNN as the backbone for defect classification. CNN effectively identifies complex patterns and structures within images. Many surface defect inspections employ various CNNs, such as ResNet [24], VGG (also VGGNet) [25], GoogLeNet [26], EfficientNet [27], MobileNet [28], ShuffleNet [29], etc. These models are pretrained on large datasets of images, allowing them to learn patterns and features indicative of defects.

Xu et al. [30] proposed the Bilinear-VGG16 model to improve quality problems that occur during the packaging process, such as packaging damage, packaging side ears opening, and scratches during the printing process. The Bilinear-VGG16 model is a network that improves performance by combining the VGG-16 network and Bilinear-CNN and uses global average pooling and global maximum pooling to extract the fine particle features of defective packaging images. This allows for robust representation learning and enhances the model’s ability to distinguish between various types of defects. They achieved an accuracy rate of the improved model recognition of 96.3%, which is better than that of the popular network models before. Zhou et al. [31] proposed a method that combines traditional computer vision (CV) techniques and deep learning models to classify all 12 types of packaging defects. This method detects some easy-to-detect defects and position offset defects using the traditional CV method, and uses ResNet-34 to detect the remaining defects. This method was verified to fulfill the defect recognition requirements through experiments on 12 types of defects. Sheng et al. [32] propose a method based on the ECA-EfficientDet to detect multiple classes of defects and solve the model’s generalization problem caused by the lack of packaging defect samples in the industry. This method improves fault detection accuracy by designing an ECA-Convblock convolutional block that can predict channel importance and suppress channels that do not carry information. Additionally, the mosaic augmentation technique and Mish activation function were used on sample data to improve the generalization function and the robustness of the model in complex environments. As a result, this method achieved an accuracy of over 99% for almost all categories except for a few.

Although these studies achieve good defect inspection results from high-quality images, none of them address the issue of low-quality images caused by both motion blur and focus blur. Thus, in this paper, we propose a technique that shows excellent inspection performance even in environments where high and low-quality (blurred) images are mixed.

## 3. Our Method

Single CNN models have the disadvantage of degrading performance when processing low-quality images, such as motion blur, and focus blur. To address this challenge, we present an inference method designated as TQE. TQE is a combination of temporal weight and quality weight. This methodology presupposes three aspects. Firstly, we deal with images that have been observed and memorized. Observed images are up-to-date images. On the other hand, memorized images are previously seen images in which prediction has already been performed and has a temporal order. Secondly, we do not process all acquired images. Generally, packaging moves gradually on the conveyor, resulting in small changes between consecutive frames. Therefore, instead of using all consecutive images, we use the small change captured in quantitative units of 2 to 4 images as input images. Lastly, low-quality images usually reduce inference performance. However, it assumes that some low-quality images may contain important defect information that high-quality images do not have. This premise is validated through experiments conducted in Section 4. The following subsection provides a detailed exposition of our method.

### 3.1. Overall Architecture

**Figure 2:**
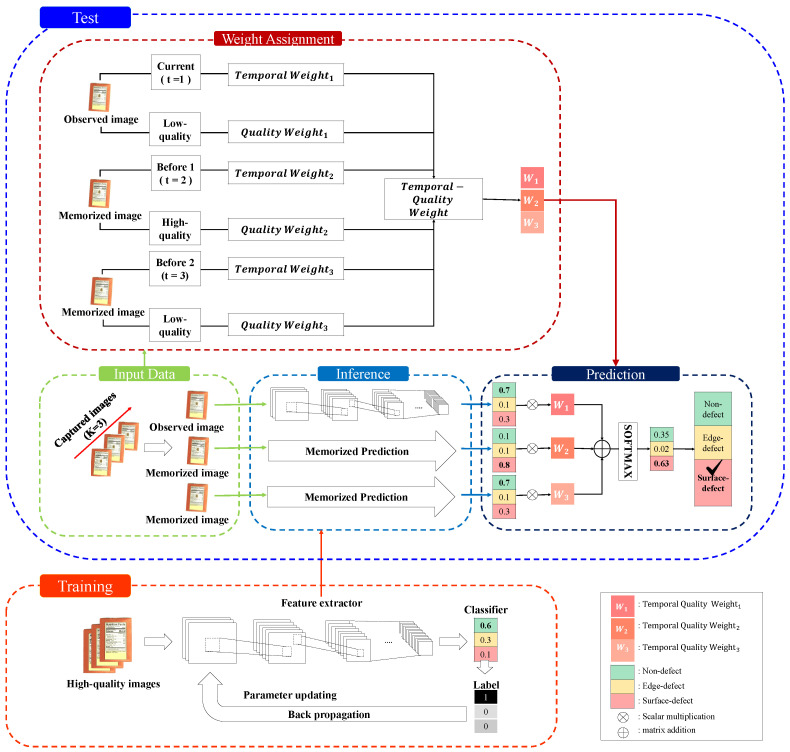
The detailed architecture of our method.

The overall architecture is shown in Figure 2. It can be largely divided into training and test parts. We aim to ensure that a CNN trained solely on high-quality images performs even on low-quality images, using TQE; thus, TQE is only used in the test part. The training part adopts the widely used CNN as the backbone network. CNNs consist of a feature extractor layer and a classifier layer, and when the probability is output by the classifier, the loss value is calculated and used to update parameters through backpropagation [33].

The test part performs predictions using single CNN models learned in the training part and TQE, which combines temporal and quality weights. As the previous premise, the image from the current moment is captured and used as an observed image, and the images from the previously captured image are loaded as memorized images. These images are selected as input data. Input data are then pass through to a pretrained model to perform inference, and at the same time, the input data are transmitted for weight allocation of TQE. TQE’s weights are determined based on the relative quality and captured order between observed and memorized images. These weights determine the final prediction by differentially applying observed and previously memorized images. These weights help the final prediction by providing different priorities to observed and previously memorized images. The following subsection provides a detailed exposition of the temporal and quality weights, which are the main components of our method.

### 3.2. Temporal Ensemble

Temporal ensemble (TE) is a method which assigns temporal weights to input images based on their timing order. This principle is widely used across various fields, where closely timed images often share similar features [34]. This means that images taken close in time tend to have similar features, allowing them to complement each other. Expanding on this idea, we introduce a temporal weighting mechanism where the importance gradually decreases or increases as the time difference between the observed image and the memorized image grows. This weighting strategy enhances temporal smoothing and integrates relevant information from observed and memorized images. Temporal weight is defined as

(1) $$TW_{t}=\frac{\exp(-|t-1|/\tau )}{\sum_{t=1}^{K}\exp\left(-\left|t-1\right|/\tau \right)}$$

where *t* is a positive integer timestamp. *K* represents the total number of images. When a new image is captured, it becomes the observed image and assigned t=1. As the next image is captured, the previously observed image (t=1) shifts as t=2 and is categorized into the memorized images. As *t* increases up to *K*, *K*-th image becomes the oldest memorized image in the sequence. This cycle repeats with each new image capture. The absolute value |t−1| is the sequence difference between the observed and memorized images. τ is a parameter that adjusts the importance of the temporal weight with positive rational numbers greater than 0. As τ approaches 0, the influence of the observed image and recent image on the temporal weight becomes significant, whereas as τ approaches *∞*, the importance of the recent and old images on the temporal weight gradually equalizes. Thereby, τ also allows for the enabling of higher weighting for recently observed images and balances the quality weights even if they are with low quality. However, τ requires user-based optimization, making it difficult to determine arbitrary numbers. Therefore, as a guideline, we present the binary search algorithm that recommends τ when the desired range of temporal weights for the observed image is inputted. The guideline for computing τ using binary search is shown in Algorithm 1.
**Algorithm 1** Binary Search for τ  1:**Input:** Target weight ($TW_{1}$), range (deviation), minimum tau ($\min_{\tau }$), maximum tau ($\max_{\tau }$), total number of input images (*K*)  2:**Output:** τ  3:Initialize t←[−iforiin[0, K]]  4:**while** true **do**  5:    Compute $\tau \leftarrow (\min_{\tau }+\max_{\tau })/2$  6:    Compute $w_{t}\leftarrow \left[\exp\left(t/\tau \right)\right]$  7:    Compute $w_{sum}\leftarrow \sum w_{t}$  8:    Compute $w_{r}\leftarrow \left[\left(x/w_{sum}\right)\;\mathrm{for}\;x\;\mathrm{in}\;w_{t}\right]$  9:    **if** $\left(TW_{1}-deviation\right)\leq w_{r}\left[0\right]\;\mathrm{and}\;w_{r}\left[0\right]\leq \left(TW_{1}+deviation\right)$ **then**10:        **break**11:    **end if**12:    **if** $w_{r}\left[0\right]<\left(TW_{1}-deviation\right)$ **then**13:        $\max_{\tau }\leftarrow \tau$14:    **else**15:        $\min_{\tau }\leftarrow \tau$16:    **end if**17:**end while**18:**return** τ

### 3.3. Quality Ensemble

Quality ensemble (QE) is a method that assigns quality weights to input images based on their sharpness. High-quality images depict sharp high-frequency features such as small details or sharp edges. In contrast, low-quality images exhibit features with blurred low-frequency details such as object boundaries or edges [35]. We use the Laplacian operator [36] to evaluate the quality of observed and previously memorized images. The Laplacian operator is a mathematical operator used to measure the spatial variation of a scalar field. It can be expressed as a combination of the gradient and divergence operators, which is defined as follows.

(2) $$\nabla^{2}f=\nabla *\left(\nabla f\right)$$

where ∇ represents the gradient operator, which quantifies the rate of change of a scalar function in space. ∇f denotes the divergence operator, measuring the extent to which a vector field diverges from a specific point. Therefore, the Laplacian operator signifies taking the gradient first and then calculating the divergence of the outcome. Essentially, the Laplacian operator assesses the spatial variation of the function, aiding in the identification of areas with significant change or curvature in the scalar field.

We evaluate the relative image quality of the observed image and the previously memorized image using Laplacian variance [37]. Initially, to calculate the Laplacian variance, we apply a Laplacian filter to transform the observed image and the previously memorized image into Laplacian images. Then, we gather Laplacian values for each pixel of the Laplacian image and compute the variance to quantify the intensity of high-frequency components in the image. Higher Laplacian variance indicates a high-quality image with high-frequency features. It is defined as

(3) $$L=\nabla^{2}I$$

where *I* is the given image, and the result of applying the Laplacian filter to this image is denoted as *L*. Here, the variance is computed as the average of the squares of the differences between each pixel’s value and the mean value. This can be expressed in the following formula:

(4) $$Var\left(L\right)=\frac{1}{n}\sum_{i=1}^{n}{\left(L_{i}-\mu \right)}^{2}$$

where $L_{i}$ is the value of the *i*-th pixel of the image after applying the Laplacian filter, μ is the mean value of all pixels *L* in the image, and *n* is the total number of pixels in the image. To calculate the variance ratio for a specific image among observed and memorized images, we divide the variance of that specific image by the sum of the variances of all images. Therefore, the variance ratio for a specific image can be calculated as follows:

(5) $$QW_{t}=\frac{Var{\left(L\right)}_{t}}{\sum_{j=1}^{K}Var{\left(L\right)}_{j}}$$

where *t* is a positive integer timestamp. *K* represents the total number of images, and $Var{\left(L\right)}_{j}$ denotes the result of applying the Laplacian filter to the *j*-th image. This is the value obtained by dividing the variance of the *t*-th image by the total variance, indicating how much the variance of the *t*-th image contributes to the overall variance of the entire image.

### 3.4. Temporal-Quality Ensemble

The proposed TQE weight considers both quality and temporal weight together. This calculates the average of the weights $TW_{t}$ and $QW_{t}$, denoted as $TQ_{t}$. It is defined as

(6) $$TQ_{t}=\frac{TW_{t}+QW_{t}}{2}$$

where *t* is a positive integer timestamp. $TW_{t}$ represents temporal weights for a specific *t*-th image, and $QW_{t}$ represents quality weights for the same *t*-th image. The algorithm for computing the proposed ensemble learning is shown in Algorithm 2.
**Algorithm 2** Temporal-Quality Ensemble Inference  1:**Input:** Total number of time stamps (*K*), collection of input images (*I*), parameter (τ)  2:**Output:** Weighted outputs from the model (*O*)  3:// *Calculate temporal weights*  4:Initialize t←[−iforiin[0, K]]  5:Compute $w_{t}\leftarrow \left[\exp\left(t/\tau \right)\right]$  6:Compute $w_{sum}\leftarrow \sum w_{t}$  7:Compute $w_{r}\leftarrow \left[\left(x/w_{sum}\right)\;\mathrm{for}\;x\;\mathrm{in}\;w_{t}\right]$  8:// *Calculate quality weights*  9:Initialize v←[]10:Initialize V←011:**for** each *i* in *I* **do**12:    Convert $g_{i}\leftarrow Gray\left(i\right)$13:    Calculate Laplacian: $\sigma_{i}\leftarrow Laplacian\left(g_{i},\;64\right).var\left(\right)$14:    Append $\sigma_{i}$ to *v*15:    Accumulate $\sigma_{i}$ to *V*16:**end for**17:Compute $v_{r}\leftarrow \left[v_{i}/V\;\mathrm{for}\;v_{i}\;\mathrm{in}\;v\right]$18:// *Calculate ensemble weights considering temporal and quality weights*19:Compute $e_{w}\leftarrow \left[\left(x+y\right)/2\;\mathrm{for}\;x,y\;\mathrm{in}\;zip\left(w_{r},v_{r}\right)\right]$20:// *Perform model inference*21:O←Model(I)22:// *Apply weighted averaging on outputs*23:$O\leftarrow O*e_{w}$24:**return** *O*

## 4. Experiments and Results

### 4.1. Datasets

**Figure 3:**
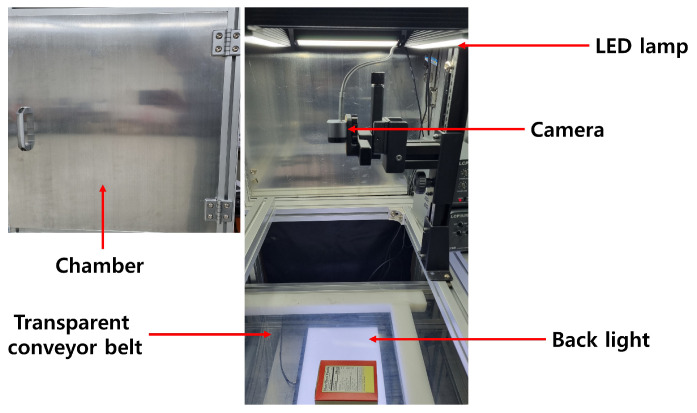
The image acquisition system has an image acquisition unit, light sources, a backlight, a transparent conveyor belt, and a chamber.

In this section, we present a comprehensive description of our collected dataset. In general, it is difficult to find an open dataset containing both high-quality and low-quality packaging images. Therefore, we collected a new dataset by acquiring data through our proprietary image acquisition system, as shown in Figure 3. The acquisition system employed a range of illumination angles and positions, along with a machine vision (BFLY-PGE-31S4C-C, FLIR, Wilsonville, OR, USA) and webcam (Brio, Logitech, Lausanne, Switzerland) with a frame rate of 10FPS. The machine vision contained a focal-length 16 mm fixed megapixel lens (LM16JC5M2, KOWA, Nagoya, Japan). The LED line illuminations were used to adjust the angle independently as bar illuminations mounted in four directions (LDBQ300, LFINE, Incheon, Republic of Korea). A backlight (LXL300, LFINE, Incheon, Republic of Korea) is mounted beneath transparent conveyors, utilizing LED mounted at regular intervals to provide a wide illumination angle and high uniformity.

**Figure 4:**
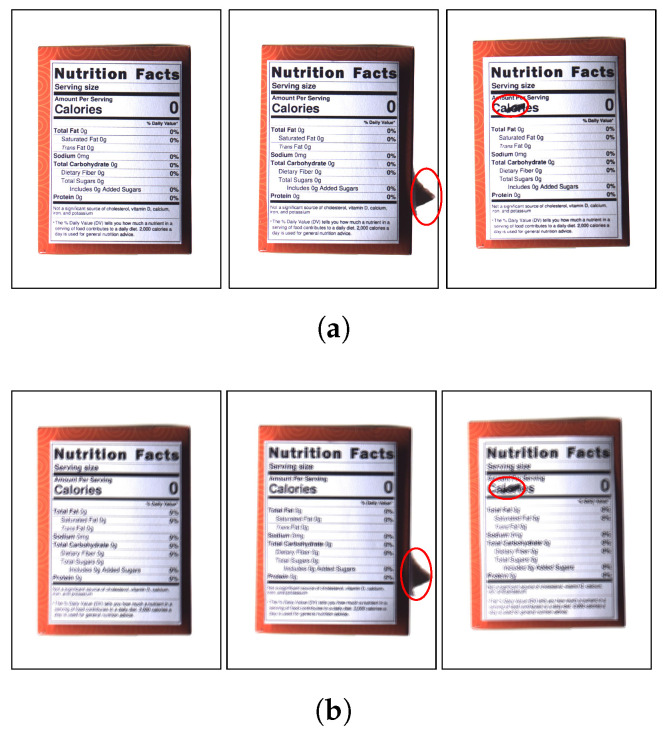
Samples of collected image. The red circles indicate defects. Left: non-defect. Middle: edge defect. Right: surface defect. (**a**) High-quality images. (**b**) Low-quality images.

To intentionally simulate motion blur issues caused by external factors, our image acquisition system was set up to capture moving packaging on a conveyor belt operating at 15 cm/s. In addition, in order to reproduce focus blur, we collected data by randomly changing the camera’s focusing function between automatic and manual modes. This configuration simulates acquiring low-quality and high-quality images, as shown in Figure 4. And Figure 4 shows the original captured image without manual cropping.

Packaging defects are classified into five categories: label loss, deformation, cracks, surface damage, and surface dirt. These categories broadly are classified into edge defects and surface defects [38]. Among them, we focus on the above two types of defects formed around product labels. This is based on the fact that the label surface of packaging inherently contains important ingredient lists, which are depicted on the left in Figure 4. For example, edge defects occur when packaging collides with other objects while traveling on a conveyor or are torn when they are improperly stacked as depicted in the middle of Figure 4. Additionally, surface defects are problems caused by ink bleeding or damage adsorbed on the label surface, as shown on the right of Figure 4.

**Figure 5:**
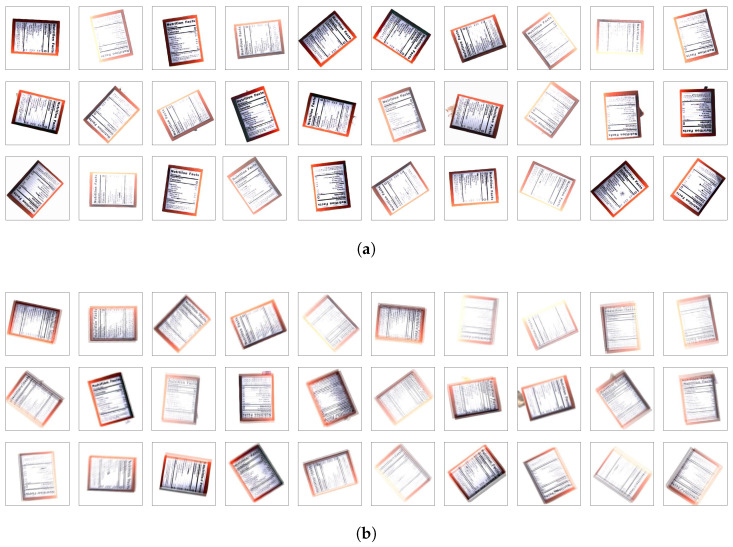
Samples of datasets. First row: non-defect. Second row: edge defect. Third row: surface defect. (**a**) High-quality images. (**b**) Low-quality images.

This experiment utilized a total of 9000 packaging images, resized to 256 × 256 pixels without cropping, and contained three classes: non-defect (3000), edge defect (3000), and surface defect (3000). Samples of the dataset are shown in Figure 5. This dataset aims to verify that CNN models trained solely on high-quality images perform effectively not only on high-quality images but also on low-quality images. For this purpose, low-quality images were selected based on having Laplacian variance values of 50% or less compared to the high-quality images. Low-quality images were collected focusing on two types of blur: motion blur (1500) and focus blur (1500), for a total of 3000 images. Each type includes three classes: non-defect (500), edge defect (500), and surface defect (500). The distribution of images in the dataset for each class is shown in Table 1.

### 4.2. Evaluation Metrics

To evaluate the performance of the proposed methods, various evaluation metrics have been used, and they are as follows:

(7) $$Precision=\frac{TP}{TP+FP}$$

(8) $$\begin{array}{c} Recall=\frac{TP}{TP+FN} \end{array}$$

(9) $$F1\;-\;Score=\frac{2TP}{2TP+FP+FN}$$

(10) $$Accuracy=\frac{TP+TN}{TP+TN+FP+FN}$$

True positive (TP) are total cases where the prediction is positive, and the actual value is positive. True negative (TN) are total cases where the prediction is negative and the actual value is negative. Conversely, False positive (FP) are total cases where the prediction is positive and the actual value is negative. False negative (FN) are total cases where the prediction is negative and the actual value is positive. Precision is the ratio of the number of classified positive to the total number of prediction positive. Recall is the ratio of the number of prediction positive to the number of actual positive. F1-Score is the balanced measure of precision and recall. Accuracy measures how often the predictions are true by comparing the classified cases to all cases.

### 4.3. Implemental Details

Ensembles based on CNN models can be used as an outstanding feature extractor for classifying tasks. In particular, CNN models trained on ImageNet [39] necessitate only minor fine-tuning, saving time and computational cost compared to training models from scratch on private datasets. Therefore, we considered the CNN models, which are capable of transfer learning through ImageNet and have proven reliability through many studies, as the baseline models. As a result, we chose ResNet-34, EfficientNet, ECAEfficientNet [40], GoogLeNet, and ShuffleNetV2 [41].

All experiments were performed on the ubuntu18.04 OS; the CPU was Intel Core i9-13900K (32-core) (Santa Clara, CA, USA), and the GPU was NVIDIA GeForce RTX 4080 (16 GB) (Santa Clara, CA, USA). We adjusted the hyperparameters listed in Table 2 throughout the model training process. We trained the network for 6 epochs. During training, we utilized the WarmupScheduler to automatically adjust the learning rate. All models were trained using AdamW, with a learning rate of 1.25 × 10^−4^ and a weight decay of 0.05.

### 4.4. Experimental Results

#### 4.4.1. High vs. Low-Quality Image Performance Using Single CNN Models

**Figure 6:**
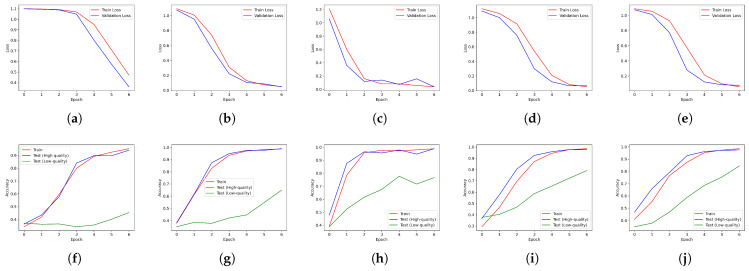
Performance analysis of CNN models on high- and low-quality images. The first row displays the loss curves for high-quality images (training and validation), while the second row shows accuracy curves for both high- and low-quality images (training, validation, and test). (**a**) ShuffleNetV2 loss. (**b**) GoogleNet loss. (**c**) ResNet-34 loss. (**d**) EfficientNet loss. (**e**) ECAEfficientNet loss. (**f**) ShuffleNetV2 accuracy. (**g**) GoogleNet accuracy. (**h**) ResNet-34 accuracy. (**i**) EfficientNet accuracy. (**j**) ECAEfficientNet accuracy.

In this experiment, the performance of CNN models was evaluated using single images of either high or low quality, without employing an ensemble. This study aimed to ascertain whether CNN models could effectively learn from private datasets and evaluate the performance disparity when using high-quality versus low-quality images as input data. The loss and accuracy curves obtained during the training of the five CNN models are shown in Figure 6. In Figure 6, the first row compares the loss convergence rates for the high-quality images. The loss was steadily decreasing with each epoch, and the validation loss was also decreasing similarly to the training loss. This showed consistent performance even on data that CNN models had not seen, indicating that the model was generalizing not only to training data but also to validation data without overfitting. The second row of Figure 6 compared the accuracy convergence rates of the CNN model on the test dataset for high- and low-quality images. In all graphs, the training accuracy consistently increased as the epochs progressed and reached over 0.9, showing stable performance, and the test accuracy also showed a similar increasing trend as the training accuracy. On the other hand, the test accuracy for low-quality images was relatively low in all graphs, but gradually increased as the epoch progressed, showing that the model was improving its adaptability even to low-quality images. However, the accuracy of the low-quality images remained at a significantly lower level than that of the high-quality images.

The class-wise results obtained on the test set of the dataset are shown in Table 3. Table 3 presents a performance comparison of CNN models on high-quality and low-quality images across various metrics. The metrics included precision, recall, F1-Score, and accuracy, with the model being evaluated on different classes: non-defect, edge defect, surface defect, and estimated total size. Analyzing the experimental results of each model for high-quality images, GoogleNet showed high precision, recall, and F1-Score in all classes, and especially recorded the highest F1-Score (0.9975) in the surface defect class. ResNet-34 also showed consistent performance in all classes, recording an F1-Score of 0.9930 in the surface defect class. ShuffleNetV2 had a precision of 0.8560 in the non-defect class, which was lower than other models, but it had the smallest model size (68.15 MB), which had the advantage of fast computation and low memory usage. On the other hand, EfficientNet and ECAEfficientNet had the largest model size (242.78 MB), showing high performance, but had the disadvantage of large memory usage. In low-quality images, the performance of all models deteriorated compared to high-quality images, and recall tended to decrease more significantly. On low-quality images, the ECAEfficientNet model showed the highest performance, with an accuracy of 0.8447. ShuffleNetV2 showed poor performance on low-quality images, especially in the non-defect class, with a recall of only 0.070, which meant it hardly detected defects in that class. Experimental results on low-quality images showed that performance on high-quality and low-quality images improved as the model size increased. In contrast, ResNet-34 maintained a relatively balanced performance with an accuracy of 0.7670, precision of 0.8079, recall of 0.7670, and F1-score of 0.7497. Additionally, from the perspective of model size, ResNet-34 had a balance. Given these attributes, ResNet-34 was selected as the representative model for other experiments, serving as a benchmark of stable, balanced performance across different image quality scenarios, and a model size that balanced efficiency with capability.

#### 4.4.2. Ablation Study on TQE: Fixed τ and Image Quality Scenarios

Table 4 compared three different ensemble techniques, AVE, TE, and QE, using ResNet-34. TE and QE were separated for TQE’s ablation analysis. In this experiment, τ was fixed to 3, and *K* represented the total number of images used in each scenario. Scenarios were focused on combinations of test images, based on high-quality (H) and low-quality (L). Furthermore, the sequence of scenarios was composed of observed images from the far left to memorized images with larger time intervals observed as we moved to the right. For example, in the HLH scenario, the observed image was of high quality, the nearest memorized image was of low quality, and the subsequent time frame presented a high-quality image. Similarly, in the LHH scenario, the observed image was low-quality, the memorized image closest in time was high-quality, and the subsequent time frame also contained a high-quality image. Among various combinations, K=3 is the ideal minimum combination to simultaneously evaluate the performance of time weight and quality weight. The reasons for choosing three combinations are as follows. Focusing on a single typed combination works the same as a single model, which negates the essence of ensembles reliant on multiple inputs. Similarly, restricting to only two combinations shows challenges in validating both temporal and quality weights simultaneously. For example, in the HL or LH combination, it is possible to evaluate temporal weighting since H and L are distinct in time. However, as the frequencies of H and L are equal, evaluating quality weighting becomes less significant. Thus, at least three combinations are required to evaluate the performance of temporal and quality weight at the same time. AVE weighted all inputs with equal importance. For example, the prediction was inaccurate in scenarios where low-quality images had the same frequency or more than high-quality images, such as HL, HLL, and HLHL scenarios. On the other hand, the TE considered the input order according to time and assigned higher weights as it approached the observed image. This method maintained relatively high performance even in scenarios with a mixture of high-quality and low-quality images, but performance tended to deteriorate in scenarios where low-quality images dominated. For example, in the HL scenario at K=2, the F1-Score showed the highest performance with 0.9900. The reason was that since H was an observed image, it was assigned a higher weight than L. Conversely, in the LH scenario, since the low-quality image was an observed image, H received a relative penalty. QE demonstrated high performance across various scenarios and provided the most consistent performance overall. In particular, it maintained good performance not only in scenarios with many high-quality images but also in scenarios with a mixture of low-quality images. Interestingly, performance tended to be better in scenarios with a mix of low-quality images than in scenarios with many high-quality images. For example, the HHL, LHH, HLHH, and LHHH scenarios showed higher performance than the H-only scenario. This may have been because it provided broader information by including images of various quality. Although QE prioritized image quality, if it included low-quality images, detailed defects or patterns that were difficult to see in high-quality images may have been more evident in the lower-quality images.

#### 4.4.3. Performance of TQE: Integrating Temporal and Quality Ensembles

Table 5 shows the performance of TQE inference methodology which combined TE and QE. Our experiment focused on combinations of test images, based on high-quality and low-quality, and was performed using various values of *K* and τ. The experimental results showed that the performance of TQE improved as the *K* and τ values increased. In detail, the experimental results were as follows. for K=2, the aggregate F1-Score at τ=1 was 0.9448, and the aggregate F1-Score at τ=3 improved to 0.9500. At τ=5, the aggregate F1-Score further improved to 0.9501. This result showed that performance improved as τ increased. For K=3, the aggregate F1-Score at τ=1 was 0.9513, which improved to 0.9537 at τ=3, and further improved to 0.9540 at τ=5. This result also showed that performance improved as τ increased. Finally, for K=4, the aggregate F1-Score at τ=1 was 0.9555, which improved to 0.9594 at τ=3, and further improved to 0.9597 at τ=5. From this, we could conclude that TQE’s performance improved in terms of aggregate performance as *K* and τ increased. The reason was that as the value of τ increased, the performance of TQE approached that of QE. τ is a factor that determines the weight of the TE. As τ increased, the proportion of QE increased and the proportion of the TE became relatively small. These results confirmed that as τ increased, the performance of TQE approached that of the QE. Particularly noteworthy among the experimental results were the LHH, LHLL, and LHHH scenarios at K=3. Despite having a low-quality image as the observed image, these scenarios outperformed the other scenarios. In the LHH scenario, when τ was 3, precision, recall, and F1-Score all recorded the highest performance of 0.9923. Additionally, in the LHLL scenario, when τ was 3, precision, recall, and F1-Score were 0.9233, 0.9160, and 0.9152, respectively, proving the effectiveness of TQE even in scenarios involving low-quality images. The LHHH scenario also showed the best performance when τ was 3, which showed a higher performance than the QE. These results showed that the TE considered temporal priority and balanced the TE with a large number of quality images without alienating a small number of quality images.

#### 4.4.4. Performance Comparison of TQE and AVE across CNN Models

**Figure 7:**
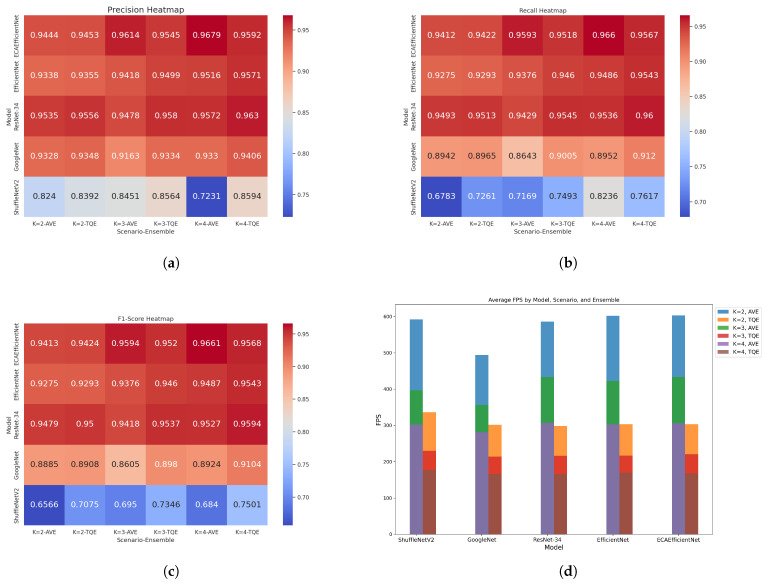
Comparison of the performance and processing speed of CNN models with the ensemble applied. (**a**) Precision heatmaps. (**b**) Recall heatmaps. (**c**) F1-Score heatmaps. (**d**) FPS bar charts.

To confirm the reliability of TQE, we applied it to five CNN models and compared them with AVE. Table 6 indicates that TQE outperforms AVE in precision, recall, F1-Score, and frames per second (FPS) across multiple models, including ShuffleNetV2, GoogleNet, ResNet-34, EfficientNet, and ECAEfficientNet. For instance, with K=2, TQE improved the F1-score of ShuffleNetV2 from 0.6566 to 0.7075 and maintained a similar trend across other models such as GoogleNet, ResNet-34, and EfficientNet. Moreover, as the value of *K* increases, the performance of the models together improves. For example, with *K* = 3, ResNet-34’s F1-score increased from 0.9418 to 0.9537, and with *K* = 4, it increased to 0.9594. Such enhancements are consistently verified in other models. The precision, recall, and F1-Score heatmaps in Figure 7 visually detail the performance enhancements, with models like ResNet-34 and ECAEfficientNet showing particularly strong improvements. However, a drawback of TQE is the decrease in FPS, when compared with AVE across all scenarios. For example, for the ShuffleNetV2 model, FPS was 336 at *K* = 2 but decreased to 230 at *K* = 3 and 177 at *K* = 4. The bar charts shown in Figure 7d visualize this drawback in that AVE maintains a higher FPS across all models and scenarios.

Table 7 shows the performance differences between TQE and single CNN models and AVE. This comparison focused on cases with at least one low-quality image, and was measured by precision, recall, and F1-score. Results showed that TQE demonstrated significant performance improvement over single CNN models across all models and scenarios. For example, in the K=2 scenario for the ShuffleNetV2 model, TQE improved by 0.0705 in precision, 0.2009 in recall, and 0.2542 in F1-score.

In comparison with AVE, TQE consistently showed better results, but the performance improvement was relatively smaller than single CNN models. This was because averaging ensembles already provided a performance boost of their own due to the way they averaged the output of multiple-quality images. TQE showed more significant performance improvement, especially at high *K* values. For example, in the ShuffleNetV2 model at K=4, recall and F1-Score improved by 0.2961 and 0.3619, respectively.

When comparing TQE and AVE with K=4, ShuffleNetV2 achieved a higher F1-score using TQE despite having a lower recall. This meant that ShuffleNetV2-based TQE made conservative predictions, resulting in low recall and high precision, and was consequently less effective at identifying all defect cases but was more accurate when it did make a prediction.

ECAEfficientNet showed higher performance in AVE than TQE as *K* increased. This was because ECAEfficientNet did not show a significant performance difference from TQE for low-quality images, even with single CNN models. In other words, because ECAEfficientNet itself showed high performance even for low-quality images, as *K* increased, the performance of AVE also increased and eventually could outperform TQE. On the other hand, for other models, single CNN models showed a large performance difference compared to TQE for low-quality images. As a result, as *K* increased, the performance of TQE became significantly better than AVE. This meant that TQE had a greater effect on networks with poor performance on low-quality images in single CNN models.

## 5. Conclusions

This paper presents TQE for identifying defects in packaging, including image blur. We conducted experiments to verify the performance of CNN models trained on high-quality images to identify defects contained in low-quality images. As a result, CNNs identified more than 94% of defects included in high-quality images, but the accuracy dropped to about 10% to 50% in low-quality images. Additionally, we conducted experiments to identify defects contained in low-quality images using ensembles. As a result, we confirmed that both AVE and TQE had better performance than the CNN model alone. However, when low-quality images comprised more than half of the input images, AVE significantly decreased the performance of CNN models. In contrast, TQE increases performance by prioritizing image quality and maintaining importance against low-quality images in temporal timing. As a result, considering cases where at least one low-quality image is included, TQE had an F1-score approximately 17.64% to 22.41% higher than single CNN models and about 1.86% to 2.06% higher than AVE. These confirm the efficiency and improvement of TQE inference, considering both low-quality and high-quality datasets, by extensively applying CNN models. Additionally, the ensemble technique which included a few low-quality images outperformed ensembles consisting solely of high-quality images only. This suggests the possibility that low-quality images provide useful features for defect inspection when included in the ensemble.

As future work, we plan to collect and analyze more types of defect patterns to improve the performance of the proposed ensemble method. In addition, we also plan to expand the scope of industrial application by redefining not only low-quality conditions caused by camera blur but also external environmental factors such as pollution and damage.

## Figures and Tables

**Table 1:** The distribution of images by class in the training, validation, and test sets of the packaging dataset.

Quality	Class	Training Set	Validation Set	Test Set	Total
High-quality images	Non-defect	1000	1000	-	2000
	Edge defect	1000	1000	-	2000
	Surface defect	1000	1000	-	2000
Low-quality images	Non-defect	-	-	1000	1000
	Edge defect	-	-	1000	1000
	Surface defect	-	-	1000	1000
Total		3000	3000	3000	9000

**Table 2:** Hyperparameter selection.

Hyperparameter	Values
Optimizer	AdamW
Loss function	Cross-entropy
Epoch	50
Batch Size	100
Learning Rate	1.25 × 10^−4^
Rate Decay	0.05

**Table 3:** Performance comparison of CNN models on a high-quality and low-quality image.

Models	Class	High-Quality Image				Low-Quality Image				Estimated Total Size (MB)
		Precision	Recall	F1-Score	Accuracy	Precision	Recall	F1-Score	Accuracy	
ShuffleNetV2	Non-defect	0.8560	0.9930	0.9194	0.9407	0.9090	0.0700	0.1299	0.4537	68.15
	Edge defect	0.9942	0.8520	0.9176		0.9040	0.2920	0.4414		
	Surface defect	0.9940	0.9770	0.9854		0.3842	0.9990	0.4536		
	Aggregate	0.9480	0.9407	0.9408		0.7324	0.4537	0.3755		
GoogleNet	Non-defect	0.9802	0.9890	0.9846	0.9893	0.9685	0.2770	0.4308	0.6487	145.02
	Edge defect	0.9919	0.9800	0.9859		0.9295	0.6720	0.7800		
	Surface defect	0.9960	0.9990	0.9975		0.5008	0.9970	0.6667		
	Aggregate	0.9894	0.9893	0.9893		0.7996	0.6487	0.6258		
ResNet-34	Non-defect	0.9919	0.9830	0.9874	0.9890	0.7525	0.9030	0.8209	0.7670	207.70
	Edge defect	0.9880	0.9850	0.9865		0.9599	0.4550	0.6174		
	Surface defect	0.9872	0.9990	0.9930		0.7112	0.9430	0.8108		
	Aggregate	0.9890	0.9890	0.9890		0.8079	0.7670	0.7497		
EfficientNet	Non-defect	0.9889	0.9810	0.9849	0.9767	0.8774	0.7370	0.8011	0.7913	242.78
	Edge defect	0.9460	0.9990	0.9718		0.6882	0.9270	0.7899		
	Surface defect	0.9979	0.9500	0.9734		0.8733	0.7100	0.7832		
	Aggregate	0.9776	0.9767	0.9767		0.8130	0.7913	0.7914		
ECAEfficientNet	Non-defect	0.9889	0.9780	0.9834	0.9753	0.9209	0.8270	0.8714	0.8447	242.78
	Edge defect	0.9407	1.0000	0.9695		0.7663	0.9180	0.8353		
	Surface defect	1.0000	0.9480	0.9733		0.8728	0.7890	0.8288		
	Aggregate	0.9765	0.9753	0.9754		0.8533	0.8447	0.8452		

**Table 4:** Comparison of ResNet-34 using AVE, TE, and QE.

Scenarios		AVE			TE			QE		
		Precision	Recall	F1-Score	Precision	Recall	F1-Score	Precision	Recall	F1-Score
*K* = 2	HH	0.9890	0.9890	0.9890	0.9890	0.9890	0.9890	0.9890	0.9890	0.9890
	HL	0.9820	0.9820	0.9820	0.9900	0.9900	0.9900	0.9854	0.9853	0.9853
	LH	0.9820	0.9817	0.9817	0.9438	0.9393	0.9389	0.9854	0.9853	0.9853
	LL	0.8610	0.8443	0.8390	0.8610	0.8443	0.8390	0.8610	0.8443	0.8390
	Aggregate	0.9535	0.9493	0.9479	0.9460	0.9407	0.9392	0.9552	0.9510	0.9497
*K* = 3	HHH	0.9890	0.9890	0.9890	0.9890	0.9890	0.9890	0.9890	0.9890	0.9890
	HHL	0.9913	0.9913	0.9913	0.9920	0.9920	0.9920	0.9917	0.9917	0.9917
	HLH	0.9913	0.9913	0.9913	0.9917	0.9917	0.9917	0.9917	0.9917	0.9917
	HLL	0.9195	0.9120	0.9108	0.9541	0.9510	0.9508	0.9652	0.9640	0.9638
	LHH	0.9913	0.9913	0.9913	0.9878	0.9877	0.9877	0.9917	0.9917	0.9917
	LHL	0.9195	0.9120	0.9108	0.9170	0.9090	0.9076	0.9652	0.9640	0.9638
	LLH	0.9195	0.9120	0.9108	0.8984	0.8883	0.8861	0.9652	0.9640	0.9638
	LLL	0.8610	0.8443	0.8390	0.8610	0.8443	0.8390	0.8610	0.8443	0.8390
	Aggregate	0.9478	0.9429	0.9418	0.9489	0.9441	0.9430	0.9651	0.9626	0.9618
*K* = 4	HHHH	0.9890	0.9890	0.9890	0.9890	0.9890	0.9890	0.9890	0.9890	0.9890
	HHHL	0.9917	0.9917	0.9917	0.9907	0.9907	0.9907	0.9907	0.9907	0.9907
	HHLH	0.9917	0.9917	0.9917	0.9910	0.9910	0.9910	0.9907	0.9907	0.9907
	HHLL	0.9820	0.9817	0.9817	0.9913	0.9913	0.9913	0.9854	0.9853	0.9853
	HLHH	0.9917	0.9917	0.9917	0.9917	0.9917	0.9917	0.9907	0.9907	0.9907
	HLHL	0.9820	0.9817	0.9817	0.9900	0.9900	0.9900	0.9854	0.9853	0.9853
	HLLH	0.9820	0.9817	0.9817	0.9865	0.9863	0.9863	0.9854	0.9853	0.9853
	HLLL	0.9015	0.8917	0.8897	0.9339	0.9280	0.9274	0.9452	0.9420	0.9415
	LHHH	0.9917	0.9917	0.9917	0.9907	0.9907	0.9907	0.9907	0.9907	0.9907
	LHHL	0.9820	0.9817	0.9817	0.9647	0.9627	0.9626	0.9854	0.9853	0.9853
	LHLH	0.9820	0.9817	0.9817	0.9438	0.9393	0.9389	0.9854	0.9853	0.9853
	LHLL	0.9015	0.8917	0.8897	0.9071	0.8977	0.8957	0.9452	0.9420	0.9415
	LLHH	0.9820	0.9817	0.9817	0.9222	0.9150	0.9139	0.9854	0.9853	0.9853
	LLHL	0.9015	0.8917	0.8897	0.8942	0.8840	0.8815	0.9452	0.9420	0.9415
	LLLH	0.9015	0.8917	0.8897	0.8816	0.869	0.8656	0.9452	0.9420	0.9415
	LLLL	0.8610	0.8443	0.8390	0.8610	0.8443	0.8390	0.8610	0.8443	0.8390
	Aggregate	0.9572	0.9536	0.9527	0.9518	0.9475	0.9466	0.9691	0.9672	0.9668

**Table 5:** Comparison of TQE based on ResNet-34 according to *K* and τ.

Scenarios		TQE								
		τ = 1			τ=3			τ = 5		
		Precision	Recall	F1-Score	Precision	Recall	F1-Score	Precision	Recall	F1-Score
*K* = 2	HH	0.9890	0.9890	0.9890	0.9890	0.9890	0.9890	0.9890	0.9890	0.9890
	HL	0.9923	0.9923	0.9923	0.9910	0.9910	0.9910	0.9897	0.9897	0.9897
	LH	0.9608	0.9590	0.9588	0.9813	0.9810	0.9810	0.9829	0.9827	0.9826
	LL	0.8610	0.8443	0.8390	0.8610	0.8443	0.8390	0.8610	0.8443	0.8390
	Aggregate	0.9508	0.9462	0.9448	0.9556	0.9513	0.9500	0.9557	0.9514	0.9501
*K* = 3	HHH	0.9890	0.9890	0.9890	0.9890	0.9890	0.9890	0.9604	0.9597	0.9604
	HHL	0.9904	0.9903	0.9903	0.9910	0.9910	0.9910	0.9914	0.9913	0.9913
	HLH	0.9917	0.9917	0.9917	0.9920	0.9920	0.9920	0.9920	0.9920	0.9920
	HLL	0.9871	0.9870	0.9870	0.9633	0.9617	0.9615	0.9572	0.9550	0.9548
	LHH	0.9861	0.9860	0.9860	0.9923	0.9923	0.9923	0.9920	0.9920	0.9920
	LHL	0.9331	0.9273	0.9265	0.9442	0.9403	0.9399	0.9454	0.9417	0.9412
	LLH	0.9116	0.9030	0.9012	0.9313	0.9253	0.9245	0.9381	0.9333	0.9327
	LLL	0.8610	0.8443	0.8390	0.8610	0.8443	0.8390	0.8610	0.8443	0.8390
	Aggregate	0.9563	0.9523	0.9513	0.9580	0.9545	0.9537	0.9583	0.9548	0.9540
*K* = 4	HHHH	0.9890	0.9890	0.9890	0.9890	0.9890	0.9890	0.9890	0.9890	0.9890
	HHHL	0.9900	0.9900	0.9900	0.9904	0.9903	0.9903	0.9904	0.9903	0.9903
	HHLH	0.9903	0.9903	0.9903	0.9910	0.9910	0.9910	0.9910	0.9910	0.9910
	HHLL	0.9910	0.991	0.9909	0.9917	0.9917	0.9917	0.9914	0.9913	0.9913
	HLHH	0.9910	0.991	0.9909	0.9913	0.9913	0.9913	0.9913	0.9913	0.9913
	HLHL	0.9923	0.992	0.9923	0.9910	0.9910	0.9910	0.9897	0.9897	0.9897
	HLLH	0.9920	0.9920	0.9920	0.9877	0.9877	0.9876	0.9868	0.9867	0.9866
	HLLL	0.9781	0.9777	0.9776	0.9403	0.936	0.9354	0.9316	0.9257	0.9248
	LHHH	0.9904	0.9903	0.9903	0.9920	0.9920	0.9920	0.9917	0.9917	0.9917
	LHHL	0.9690	0.9680	0.9679	0.9850	0.9850	0.9850	0.9858	0.9857	0.9856
	LHLH	0.9608	0.9590	0.9588	0.9810	0.9810	0.9810	0.9829	0.9827	0.9826
	LHLL	0.9182	0.9107	0.9093	0.9233	0.9160	0.9152	0.9228	0.9157	0.9145
	LLHH	0.9382	0.9333	0.9327	0.97149	0.97067	0.97055	0.9797	0.9793	0.9793
	LLHL	0.9043	0.8947	0.8926	0.91349	0.9050	0.90332	0.9166	0.9087	0.9072
	LLLH	0.8979	0.8873	0.8849	0.90757	0.8983	0.89647	0.9119	0.9033	0.9016
	LLLL	0.8610	0.8443	0.8390	0.8610	0.8443	0.8390	0.8610	0.8443	0.8390
	Aggregate	0.9596	0.9563	0.9555	0.9630	0.9600	0.9594	0.9634	0.9604	0.9597

**Table 6:** Test result of five CNN models under AVE and TQE.

Scenarios	Models	AVE				TQE			
		Aggregate				Aggregate			
		Precision	Recall	F1-Score	FPS	Precision	Recall	F1-Score	FPS
*K* = 2	ShuffleNetV2	0.8240	0.6783	0.6566	592	0.8392	0.7261	0.7075	336
	GoogleNet	0.9328	0.8942	0.8885	494	0.9348	0.8965	0.8908	301
	ResNet-34	0.9535	0.9493	0.9479	586	0.9556	0.9513	0.9500	298
	EfficientNet	0.9338	0.9275	0.9275	602	0.9355	0.9293	0.9293	303
	ECAEfficientNet	0.9444	0.9412	0.9413	603	0.9453	0.9422	0.9424	303
*K* = 3	ShuffleNetV2	0.8451	0.7169	0.6950	397	0.8564	0.7493	0.7346	230
	GoogleNet	0.9163	0.8643	0.8605	356	0.9334	0.9005	0.8980	214
	ResNet-34	0.9478	0.9429	0.9418	433	0.9580	0.9545	0.9537	216
	EfficientNet	0.9418	0.9376	0.9376	422	0.9499	0.9460	0.9460	217
	ECAEfficientNet	0.9614	0.9593	0.9594	433	0.9545	0.9518	0.9520	221
*K* = 4	ShuffleNetV2	0.7231	0.8236	0.6840	302	0.8594	0.7617	0.7501	177
	GoogleNet	0.9330	0.8952	0.8924	281	0.9406	0.9120	0.9104	166
	ResNet-34	0.9572	0.9536	0.9527	307	0.9630	0.9600	0.9594	166
	EfficientNet	0.9516	0.9486	0.9487	303	0.9571	0.9543	0.9543	170
	ECAEfficientNet	0.9679	0.9660	0.9661	306	0.9592	0.9567	0.9568	168

**Table 7:** The difference between the performance of TQE and other methods, considering cases where at least one low-quality image is included.

Scenarios	Models	Difference (TQE–Single CNN Model)			Difference (TQE–AVE)		
		Aggregate			Aggregate		
		Precision	Recall	F1-Score	Precision	Recall	F1-Score
*K* = 2	ShuffleNetV2	0.0705	0.2009	0.2542	0.0203	0.0637	0.0679
	GoogleNet	0.1170	0.2169	0.2322	0.0027	0.0031	0.0031
	ResNet-34	0.1366	0.1717	0.1873	0.0028	0.0027	0.0028
	EfficientNet	0.1085	0.1222	0.1221	0.0023	0.0024	0.0024
	ECAEfficientNet	0.0816	0.0865	0.0862	0.0012	0.0013	0.0015
	Aggregate	0.1028	0.1596	0.1764	0.0058	0.0146	0.0155
*K* = 3	ShuffleNetV2	0.1109	0.2683	0.3296	0.0129	0.0370	0.0453
	GoogleNet	0.1258	0.2391	0.2592	0.0195	0.0414	0.0429
	ResNet-34	0.1457	0.1826	0.1990	0.0117	0.0133	0.0136
	EfficientNet	0.1329	0.1503	0.1502	0.0093	0.0096	0.0096
	ECAEfficientNet	0.0981	0.1037	0.1035	−0.0079	−0.0086	−0.0085
	Aggregate	0.1227	0.1888	0.2083	0.0091	0.0185	0.0206
*K* = 4	ShuffleNetV2	0.1211	0.2961	0.3619	0.1454	−0.0660	0.0705
	GoogleNet	0.1377	0.2581	0.2793	0.0081	0.0179	0.0192
	ResNet-34	0.1534	0.1911	0.2077	0.0062	0.0068	0.0071
	EfficientNet	0.1427	0.1615	0.1614	0.0059	0.0061	0.0060
	ECAEfficientNet	0.1047	0.1108	0.1104	−0.0093	−0.0099	−0.0099
	Aggregate	0.1319	0.2035	0.2241	0.0313	-0.0090	0.0186

## Data Availability

The data presented in this study are available on request from the corresponding author.
